# The isolation of VCAM-1^+^ endothelial cell-derived extracellular vesicles using microfluidics

**DOI:** 10.20517/evcna.2023.51

**Published:** 2024-02-08

**Authors:** Naveed Akbar, Evelyn Grace Luciani, Raheel Ahmad, Dasol Lee, Sara Veiga, Daniel Christopher Rabe, Shannon Leigh Stott

**Affiliations:** ^1^Division of Cardiovascular Medicine, Radcliffe Department of Medicine, University of Oxford, Oxford OX3 9DU, UK.; ^2^Center for Engineering in Medicine & Surgery, Massachusetts General Hospital Cancer Center, Harvard Medical School, Charlestown - Boston, MA 02129, USA.

**Keywords:** Cardiovascular, vascular, biomarker, inflammation, atherosclerosis, endothelium

## Abstract

**Background:** Vascular cell adhesion molecule-1 (VCAM-1^+^) endothelial cell-derived extracellular vesicles (EC-EVs) are augmented in cardiovascular disease, where they can signal the deployment of immune cells from the splenic reserve. Endothelial cells in culture activated with pro-inflammatory tumor necrosis factor-α (TNF-a) also release VCAM-1^+^ EC-EVs. However, isolating VCAM-1^+^ EC-EVs from conditioned cell culture media for subsequent in-depth analysis remains challenging.

**Aim:** We utilized the extracellular vesicles (EV) microfluidics herringbone chip (^EV^HB-Chip), coated with anti-VCAM-1 antibodies, for selective capture of VCAM-1^+^ cells and EC-EVs.

**Methods and Results:** Engineered EA.hy926 endothelial cells overexpressing VCAM-1 (*P* < 0.001 versus control) showed increased binding to the VCAM-1- ^EV^HB-Chip versus an IgG device. TNF-α-stimulated human umbilical cord vein endothelial cells (HUVECs) exhibited elevated VCAM-1 protein levels (*P* < 0.001) and preferential binding to the VCAM-1- ^EV^HB-Chip versus the IgG device. HUVECs stimulated with TNF-α showed differential gene expression of intercellular adhesion molecule-1 (ICAM-1) (*P* < 0.001) and VCAM-1 (*P* < 0.001) by digital droplet PCR versus control cells. HUVEC-derived EC-EVs were positive for CD9, CD63, HSP70, and ALIX and had a modal size of 83.5 nm. Control and TNF-α-stimulated HUVEC-derived EC-EV cultures were captured on the VCAM-1- ^EV^HB-Chip, demonstrating selective capture. VCAM-1^+^ EC-EV were significantly enriched for ICAM-1 (*P* < 0.001) mRNA transcripts.

**Conclusion:** This study presents a novel approach using the ^EV^HB-Chip, coated with anti-VCAM-1 antibodies and digital droplet PCR for the study of VCAM-1^+^ EC-EVs. Isolation of VCAM-1^+^ EC-EV from heterogeneous sources such as conditioned cell culture media holds promise for subsequent detailed characterization, and may facilitate the study of VCAM-1^+^ EC-EVs in cardiovascular and metabolic diseases, for disease monitoring and therapeutic insights.

## INTRODUCTION

Endothelial cell activation is a hallmark feature of cardiovascular and metabolic diseases. Endothelial cell activation is marked by the transition of the endothelium from a quiescent non-adherent state to a pro-inflammatory phenotype. In patients, this results in a concomitant attenuation of endothelial cell vasodilator function, which is an independent risk factor for cardiovascular diseases.

Expression of vascular cell adhesion molecule-1 (VCAM-1) is a cardinal feature in endothelial cell activation, which plays a pivotal role in immune cell recruitment to local sites of inflammation^[1]^. VCAM-1 is a glycoprotein and can be upregulated in response to pro-inflammatory cytokines, including tumor necrosis factor-α (TNF-α) on the luminal surface of blood vessels. VCAM-1 facilitates immune cell binding to the endothelial lining through its interaction with receptors such as very late antigen-4 (VLA-4), which are prominently expressed on immune cell populations, including neutrophils and monocytes.

Endothelial cell activation and VCAM-1 expression also signal across-organ deployment of immune cells from remote reservoirs, such as the spleen, following acute myocardial infarction (MI) via endothelial cell-derived extracellular vesicles (EC-EVs). EC-EVs are elevated in the peripheral blood of patients as they present to the hospital. Plasma EV concentrations following MI correlate with the extent of myocardial injury immediately after MI, the size of the resulting cardiac scar six months post-MI, and EC-EV-VCAM-1^+^ correlates with the magnitude of the peripheral blood neutrophil response following MI^[2]^. Blood neutrophil counts are an independent risk factor for mortality and diminished cardiac function post-MI in clinical cohorts^[3]^. EC-EVs are specialized membrane-closed vesicles that harbor genetic material, lipids, and protein. Once liberated to the peripheral blood, VCAM-1^+^EC-EVs rapidly localize to various organs, including the liver and spleen, where they facilitate the mobilization and transcriptional activation of splenic monocytes and splenic neutrophils, priming them for subsequent recruitment to the heart. Significantly, the genetic manipulation of mouse endothelial cells using CRISPR/Cas9-based editing techniques has enabled the generation of VCAM-1-deficient EC-EVs, even after inflammatory stimulation. These VCAM-1 knock-out EC-EVs fail to deploy splenic-immune cells, unlike wild-type VCAM-1 rich EC-EV, providing valuable insights into the cross-organ communication of EC-EV following MI^[2]^. EC-EV enrichment following activation of endothelial cells is not limited to MI; similar observations are made in metabolic diseases^[4]^, diabetes, and pre-clinical models of stroke and traumatic brain injury^[5]^. EC-EV-mediated cross-organ communication extends to the liver following injury to the brain, where it induces Kupffer cell activation, triggers the acute phase response, and mediates sickness behavior in otherwise healthy rodents^[6]^. Cultured human and mouse endothelial cells stimulated with pro-inflammatory TNF-α also display this enrichment for EC-EV-VCAM-1 on their EV-surface, mediate monocyte and macrophage chemokinesis, and enhance chemotaxis to macrophage chemoattractant protein-1 (MCP-1)^[7]^.

Despite the expanding interest in EC-EVs and their role in intercellular communication, the isolation of specific subpopulations of EC-EVs, such as those that express VCAM-1, remains challenging, particularly when targeting low-abundance molecules. Existing immunoaffinity techniques using commonly employed extracellular vesicle (EV) markers, such as CD9, CD63, and CD81, have demonstrated limitations in providing the requisite specificity for diverse EV populations^[8]^. To address these challenges, recent advancements in microfluidic technology, such as the herringbone (HB)-Chip, originally optimized for capturing rare circulating tumor cells and low-abundance EV (^EV^HB-Chip) populations from patient plasma, have emerged as a promising tool. Innovations in aryl diazonium fabrication of plastic and nanoparticle-based modifications have facilitated the specificity of the Herringbone device surface^[9]^.

Here, we present the utilization of the VCAM-1- ^EV^HB-Chip as an innovative method for capturing VCAM-1^+^ cell populations and VCAM-1^+^ EC-EV, providing a powerful tool for the differential analysis of these biologically active vesicles. This approach may enable the mechanisms underlying EC-EV-mediated immune cell deployment, transcriptional programming, and recruitment in vascular inflammation to be studied at unprecedented depths, offering novel insights that may have far-reaching implications in the field of cardiovascular and metabolic diseases.

## MATERIALS AND METHODS

### Cell culture

EA.hy926 cells (ATCC, CRL-2922) were cultured in a Dulbecco's Modified Eagle's Medium (4,500 mg/L glucose and sodium bicarbonate and sodium pyruvate, liquid, sterile-filtered) (Sigma, D5671-500ML) supplemented with 10% heat-inactivated fetal bovine serum (FBS) (US origin) (Thermosteric, 10082147) and 1% Penicillin-Streptomycin-Glutamine (100 X) (Sigma, 10378016). Pooled human umbilical cord vein endothelial cells (HUVECs) (Lonza, P1087) were cultured in EGM^TM^-2 BulletKit^TM^ (Lonza, CC-3162) containing 2% FBS. Primary cells were cultured between passages 2 to 8. All cultures were maintained in a humidified CO_2_ incubator at 37 °C.

### VCAM-1 plasmid

Precision LentiORF VCAM1 w/ Stop Codon (Horizon Discovery, Clone: PLOHS_100067018) was purchased as a glycerol stock of E. coli containing lentiviral plasmids. Terrific Broth (Thermofisher, A1374301) containing ampicillin (100 ug/mL) (Sigma) was inoculated overnight at 37 °C with agitation (250 rounds per minute (RPM)). Plasmid DNA was isolated using ZymoPURE II Plasmid Midiprep Kit (Zymo Research, D4201) using the manufacturer’s instructions. Isolated plasmid DNA concentration was measured by spectrophotometry and stored at -80 °C.

### Endothelial cell stimulation

HUVECs were treated with recombinant human tumor necrosis factor-α (TNF-α) (Biotechne, 210-TA-020/CF) at a concentration of 10 ng/mL for the described time points.

### Lentiviral transductions

EA.hy926 cells were sequentially transduced with a palmitoylated-tdTomato fluorescent reporter^[10]^ and LentiORF VCAM-1 using lentivirus. The lentiviral constructs were based on third-generation lentivirus technology and produced in compliance with Biosafety Level 2 (BL2+) guidelines as approved by the Massachusetts General Brigham Institutional Biosafety Committee. The lentiviral packaging plasmids pMDLg/pRRE, pRSV-Rev, and pVSV-G were co-transfected with the pCSCGW2-PalmtdTomato lentiviral vector^[10]^ into HEK-293T cells, following the manufacturer's recommended protocol and employing TransIT-Lenti (Mirrus, MIR6600) as the transfection reagent. After 48 h, lentiviral supernatant was harvested and subsequently filtered through a 0.45 µm filter. TransduceIT Transduction Reagent (Mirrus, MIR6620) was used to facilitate transduction into EA.hy926 cells, followed by a 24-h incubation period. Post-transduction, cells expressing the palmitoylated-tdTomato reporter were selectively identified using flow cytometry and targeted with LentiORF VCAM-1 using lentivirus in a similar manner.

### ELISA

VCAM-1/CD106 DuoSet ELISA (Biotechne, DY809-05) with DuoSet ELISA Ancillary Reagent Kit 2 (Biotechne, DY008) were used to determine VCAM-1 protein concentrations in cell culture supernatants as described in the manufacturer’s instructions.

### Extracellular vesicle generation

HUVEC-derived EC-EVs were generated under control and TNF-α inflammatory conditions as previously described, using a validated EV method, which complies with recommendations for the study of EV by the International Society for Extracellular Vesicles^[2,7,11]^. 2 × 10^6^ HUVECs were added to a T75 cm^2^ in 10 mL of BulletKitTM (Lonza, CC-3162) media and incubated at 37 °C in a CO_2_ incubator for 24 h. When cell cultures were 80% confluent, cell culture supernatants were aspirated from the flasks. The cell monolayers received 15 mL PBS, which was left on the cells for 3 min at room temperature and then aspirated and discarded. Subsequently, 15 mL BulletKitTM (Lonza, CC-3162) media containing 2% Gibco Exosome-Depleted FBS (Thermo Fisher Scientific) from (GIBCO) with vehicle or recombinant human TNF-α 10 ng/mL was incubated with the cell monolayers for 18 h. EV-enriched supernatants were harvested and centrifuged for 5 min at 500 × *g* at 4 °C and subsequently centrifuged again at 2,000 × *g* for 10 min at 4 °C. Cleared supernatants were transferred to Amicon® Ultra-15 Centrifugal Filter Units (Millipore, UFC901024) at 4000 × *g* for 15 min at room temperature and concentrated to ~500 µL. Concentrated cell culture supernatants were subject to size exclusion chromatography (SEC) using the Izon 70 nm columns (qEVoriginal/70 nm, IZON SCIENCE LTD, New Zealand). Based on the literature^[12]^ and product specifications, these steps omit the need to utilize a membrane filter for pre-clearing cell culture supernatants for cells, cell debris, and apoptotic bodies and maintain a size-agnostic approach to EV-biomarker studies.

### Western blotting

HUVEC cell pellets and EC-EVs were lysed and vortexed with 1X RIPA buffer (Bio-Rad) supplemented with 1X protease inhibitors (Roche). For each sample, ~5 µg of protein was loaded after combining with Laemmle sample buffer (1X final concentration) containing 2-mercaptoethanol (350 mM) as a reducing agent. Samples were heated to 95 °C for 5 min.

Samples were loaded onto a 4%-20% precast polyacrylamide gel (Mini-PROTEAN® TGX™, Bio-Rad) and then transferred to methanol-activated polyvinylidene difluoride (PVDF) membranes and blocked for 1 hour with 5% skimmed milk dissolved in 1X Tris-buffered saline containing 0.1% Tween® 20 (TBST). Membranes were incubated overnight with primary antibodies (1:500 dilutions in 5% BSA with 0.02% sodium azide): Alix (Cat. # 92880, Cell Signalling), HSP70 (Cat. # 4872, Cell Signalling), CD63 (Cat. # NB100-77913, Novus Biologicals) and CD9 (Cat. # 312102, BioLegends). Subsequently, the membranes were washed three times with TBST buffer and incubated for one hour with HRP-bound secondary antibodies (1:5,000 dilutions): Anti-rabbit IgG (Cat. # 7074), Anti-mouse IgG (Cat. # 7076), depending on the source of the primary antibody. Membranes were again washed three times with TBST buffer before incubation with enhanced chemiluminescent substrates (ECL Western Blot Substrates, ThermoFisher Scientific). Images were acquired in the darkroom using autoradiography film (Hyblot Cl Autoradiography Film, Thomas Scientific).

### Nanoparticle tracking analysis

To measure the size and concentration of HUVEC EC-EVs. The sample was diluted with filtered 1X PBS (pH 7.4) at a ratio of 1:20 (v/v). The diluted sample was then filled into the Nanosight viewing unit using a 1-mL syringe (1-mL BD™ Slip Tip Syringe sterile). The Nanosight settings were adjusted according to the manufacturer's software manual (NanoSight LM10 and NTA 3.4, Malvern, UK): The camera was ramped up until particles were distinctly visible, and the detection threshold was set between 5 and 10. Three measurements were taken at different time points and a 30-second video was recorded for each measurement, which was then analyzed using the integrated NanoSight software NTA 3.4.

### Microfluidic device fabrication and functionalization

Microfluidic devices with a staggered herringbone design^[13]^ were produced, inspected, and surface-modified in accordance with previously established protocols^[14]^. Briefly, the device consists of a 1 × 3 slide with 8-channel microfluidics, fabricated using plastic micro-injection molding of cyclic olefin copolymer (COC) (thinXXS Microtechnologies)^[14]^. The height of the device channel is 50 µm. The height of the herringbone groves is expressed as a ratio to the total height of the device and set to 0.8; the axis of the channel is (θ) 45° the principal wave vector q = 2π / 100 µm. Individual herringbone groves are 50 nm apart. The device surface was functionalized using aryl-diazonium salts to enhance immunoaffinity capture^[9]^. This microfluidic device, possessing a single inlet and outlet, is commonly referred to as the “^EV^HB-Chip” (short for Extracellular Vesicle Herringbone Chip). Herringbone chips underwent thorough inspection for debris and imperfections using light microscopy. A solution containing 20 mM p-phenylenediamine (Sigma, P6001) in 1 M hydrochloric acid (HCl, Sigma, 258148), with 20 mM sodium nitrite (Sigma, 237213), was allowed to react with EZ-link biotin-NHS-ester (final concentration of 10 mM) (Pierce, 20217) for 30 min at room temperature, yielding a biotin aryl-diazonium salt. Subsequently, the devices were flushed with the biotin aryl-diazonium solution through the inlet and exposed to ultraviolet (UV) light using a UV light bed (UVP 95042001) for 10 min. Afterward, the devices were flushed with ethanol (EtOH, Sigma, 493546) to eliminate bubbles, followed by phosphate buffered saline (PBS) (Corning, MT21040CV) through the inlet. An additional volume of biotin aryl-diazonium solution was passed through the outlet of each device, followed by another 10-min ultraviolet light exposure. The devices were then flushed with ethanol to remove any remaining bubbles and then air was passed through the devices with a syringe to facilitate drying. Following production, quality control assays were conducted to affirm a uniform surface medication^[9]^ and the devices were stored at 25°C in a vacuum desiccator for up to one month. Prior to use, the devices were flushed with 0.01667% solution of 0.09 µM streptavidin nanoparticles (Spherotech, SVP01-008-5) in PBS through the inlet. Following a 15-min incubation, streptavidin nanoparticles were also introduced through the outlet of the device. The devices were incubated with the nanoparticles overnight at 4 °C for subsequent use (see below) or stored for up to one week at 4 °C.

### VCAM-1 antibody Biotinylation and VCAM-1 Device Preparation

For each chip, anti-VCAM-1 antibody (Biotechne, BBA5) and of an IgG control equivalent (Biotechne, MAB002) were used for capture experiments. Antibodies were prepared using a 2-h incubation at room temperature with rotation, in the presence of Biotin PEG SCM 2 kDa (Creative PEGworks, PJK-1900), at a molar ratio of 20:1 for biotin linker to antibody. Excess biotin linker was removed using Zeba Desalting Columns (Thermo Scientific, 89882). The resulting biotinylated antibodies were aliquoted for single use and stored at -80 °C.

Biotinylated antibodies (8 µg/mL) were introduced into the inlet of each device and incubated at room temperature for 30 min. Subsequently, the same antibody solution was flowed through the outlet of each device. Following an additional 30-minute incubation, the devices were blocked by introducing Intercept (TBS) Blocking Buffer (LICOR, 927–60001).

### VCAM-1 Device Cell capture and EV capture

EA.hy926 TdTomato-VCAM-1 expressing cells and EA.hy926 TdTomato cells were flowed over the VCAM-1 Herringbone and IgG device. For EV capture experiments, 500 µL of concentrated conditioned media was flowed through each VCAM-1 or IgG device. 500 µL of conditioned cell culture media was flowed through the herringbone capture devices at a rate of 1 mL/h. Subsequently, the devices were washed with 1.5 mL of PBS, which flowed through at a rate of 1.5 mL/h.

### Microscopic imaging

The herringbone device is sealed with a thin (170 µm; coverslip thickness) TOPAZ layer, allowing for direct imaging of captured cells and EVs. For cell imaging, a nuclear stain (DAPI) was added to the inlet port using a syringe pump, flowing at 2.5 mL/hr, followed by a PBS rinse. To image cells captured within the device, a widefield fluorescence microscope (Nikon 90i; 60X ELWD PlanApomat, Japan) was used, acquiring images with a 2× coupler attached to a CCD camera (Retiga 2000). Images were collected at the top plane of the device (the top plane of the herringbone grooves).

### RNA extraction

RNA extraction was performed on cells and extracellular vesicles captured to VCAM-1 and IgG Herringbone devices using the MagMAX mirVana Total RNA Isolation Kit (Applied Biosystems, A27828). Specifically, for each Herringbone device, a mixture comprising Lysis Buffer (from A27828), Isopropanol (Fisher Chemical, A451SK-1), and β-mercaptoethanol (Sigma Aldrich, M3148) was passed through the device by two connected syringes to the inlet and exit ports of the devices. Subsequently, RNA isolation with DNase treatment was carried out following the manufacturer's recommended manual extraction protocol (Applied Biosystems, A27828).

### Reverse transcription and ddPCR

RNA quantification was conducted employing the 1-Step RT-ddPCR Advanced Kit for Probes (Bio-Rad, 1864021) in conjunction with pre-designed primer/probe sets for individual genes, procured from Integrated DNA Technologies (IDT) [Supplementary Table 1]. The reactions were prepared by adding RNA to each reaction, along with 1,000 nM of primers (final concentration), maintaining a primer:probe ratio of 4:1. The workflow encompassed droplet generation using the QX200 AutoDG, PCR amplification performed on the C1000 Touch Thermal Cycler, droplet analysis conducted with the QX200 Droplet Reader, and data analysis facilitated by the QX Manager software (Bio-Rad).

### Schematic illustrations

Created with BioRender.com.

### Statistical analysis

Data are plotted as group means with standard deviation (SD). Normality of data was tested by the Kolmogorov-Smirnov test. For paired analysis for two independent groups, unpaired Student’s *t*-test was used. For more than two independent groups, two-way ANOVA with Šídák's multiple comparisons test correction was used. All data analysis was performed in (Graphpad Prism). *P* < 0.05 was regarded as significant.

## RESULTS

### VCAM-1+ Cell capture using the Herringbone device

**Figure 1:**
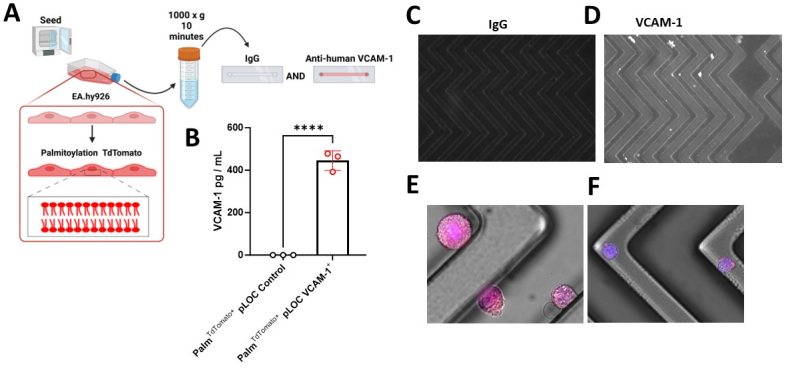
VCAM-1^+^ Endothelial Cell Capture on the VCAM-1- ^EV^HB-Chip. (A) Schematic of Experiment; (B) VCAM-1 enzyme-linked immunosorbent assay (ELISA) results for cell culture supernatants from Palm-TdTomato pLOC control and Palm-TdTomato pLOC-VCAM-1 overexpressing cell lines, presented in picograms per milliliter (pg/mL). The data represent the means ± standard deviation (SD) of three independent experiments per group. Group differences were statistically analyzed using a Student's *t*-test. The significance level is denoted as ****P < 0.0001; (C) Representative microscope image of IgG- ^EV^HB-Chip as a control; (D-F) The white signal is the fluorescence of VCAM-1^+^ endothelial cells captured on the VCAM-1- ^EV^HB-Chip surface. The white signal/dots are DAPI-stained Palm-TdTomato pLOC-VCAM-1 overexpressing cells captured on the VCAM-1- EVHB-Chip surface; (E and F) are zoomed fluorescence color images of TdTomato pLOC-VCAM-1 overexpressing cells captured on the VCAM-1- EVHB-Chip surface.

To investigate the efficacy of our VCAM-1- ^EV^HB-Chip in capturing VCAM-1^+^ cells, we initially focused on EA.hy926 cells engineered to express TdTomato-palmitoylation-VCAM-1 [Figure 1A]. Notably, these modified cells exhibited a substantial increase in the production of soluble VCAM-1 protein in their cell culture supernatants compared to their TdTomato-expressing counterparts (*P* < 0.001) [Figure 1B]. Importantly, when flowed through the VCAM-1- ^EV^HB-Chip, EA.hy926 Td-Tomato-VCAM-1 cells exhibited a robust affinity for the device's surface, while negligible binding was observed with IgG control devices [Figure 1C-F].

### Capture of HUVECs on the VCAM-1 Herringbone device with differential gene expression

**Figure 2:**
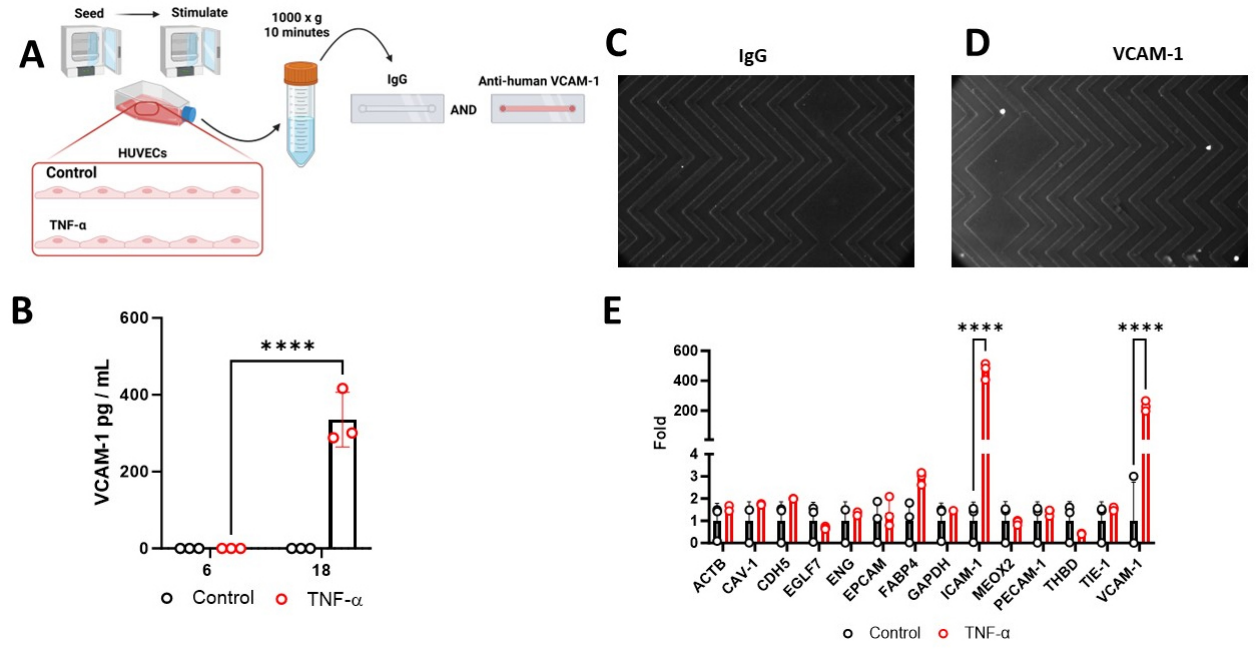
Primary Endothelial Cell Activation with Pro-inflammatory TNF-α and Capture to the VCAM-1- ^EV^HB-Chip. (A) Schematic of Experiment; (B) VCAM-1 enzyme-linked immunosorbent assay (ELISA) conducted on cell culture supernatants from human umbilical cord vein endothelial cells (HUVECs) under control and pro-inflammatory TNF-α conditions. Measurements were taken after 6 and 18 h of stimulation, with results presented as picograms per milliliter (pg/mL). The data represent the means ± standard deviation (SD) of three independent experiments per group. Statistical analysis was performed using a Student's t-test. The significance level is denoted as ****P < 0.0001; (C) representative microscope image of IgG- ^EV^HB-Chip as a control; (D-F) VCAM-1^+^ endothelial cells captured to the VCAM-1- ^EV^HB-Chip. The white signal/dots are DAPI-stained TNF-α stimulated HUVECs captured on the VCAM-1- ^EV^HB-Chip; (E) Digital droplet PCR (ddPCR) analysis of control and TNF-α stimulated HUVECs. Data are based on three independent experiments per group. The significance level is denoted as ****P < 0.0001.

To explore the VCAM-1-Herringbone’s capabilities in a physiological setting, we tested primary human umbilical cord vein endothelial cells (HUVECs) [Figure 2A]. In response to the pro-inflammatory cytokine TNF-α, these cells exhibited a significant enrichment of VCAM-1 in their culture supernatants (*P* < 0.001) [Figure 2B]. Inflammatory-activated HUVECs displayed a strong adherence to the surface of the VCAM-1 Herringbone device versus the IgG control device [Figure 2C and D]. Further analysis revealed that TNF-α-stimulated HUVECs demonstrated significant enrichment in the expression of endothelial cell activation markers, specifically VCAM-1 (*P* < 0.001) and ICAM-1 (*P* < 0.001), while the levels of other endothelial cell-associated markers remained unaffected [Figure 2E].

### Capture of VCAM-1+ HUVEC-derived extracellular vesicles (EC-EVs) on the VCAM-1 Herringbone device with differential gene expression

**Figure 3:**
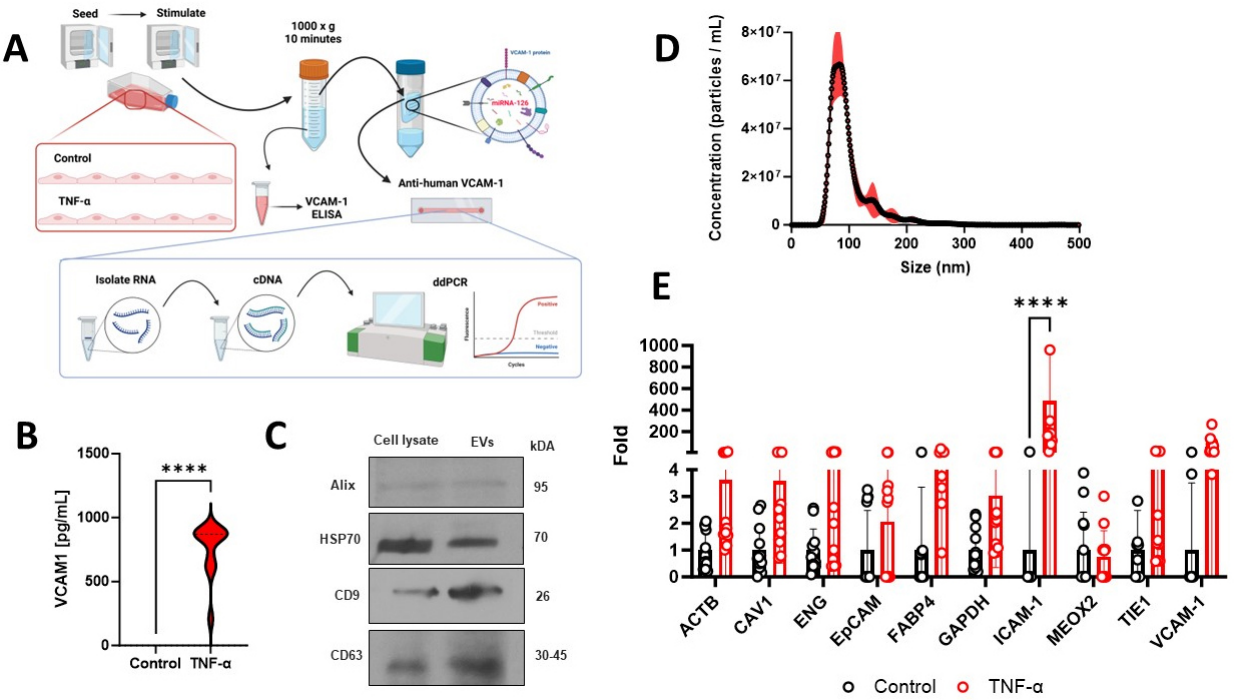
Pro-inflammatory TNF-α Activation of Primary Endothelial Cells for Capture of VCAM-1+ Endothelial Cell-Derived Extracellular Vesicles on the Herringbone Device. (A) Schematic of Experiment; (B) displays the results of the VCAM-1 enzyme-linked immunosorbent assay (ELISA) conducted on cell culture supernatants from human umbilical cord vein endothelial cells (HUVECs) under control and pro-inflammatory TNF-α conditions. Measurements were taken after 6 and 18 h of stimulation, with results presented as picograms per milliliter (pg/mL). The data represent the means ± standard deviation (SD) of twelve independent experiments per group. The significance level is denoted as ****P < 0.0001; (C) HUVEC-derived EC-EVs are positive for CD9, CD63, HSP70 and ALIX. Cell lysate is a HUVEC cell pellet; (D) Nanoparticle Tracking Analysis of HUVEC-derived EC-EVs; (E) Digital droplet PCR (ddPCR) analysis of control and TNF-α stimulated HUVEC-derived extracellular vesicles captured on the VCAM-1- ^EV^HB-Chip. The significance level is denoted as ****P < 0.0001.

Building upon our findings with HUVECs, we explored the capture of VCAM-1^+^ EC-EVs derived from inflammatory-activated cells [Figure 3A]. Consistent with previous observations, HUVECs exposed to TNF-α exhibited a significant increase in VCAM-1 levels within their cell culture supernatants [Figure 3B] HUVEC-derived EC-EVs were positive for CD9, CD63, HSP70, and ALIX [Figure 3C and Supplementary Figure 1] and had a modal size of 83.5 nm by Nanoparticle Tracking Analysis [Figure 3D]. Subsequent analysis of VCAM-1+ EC-EVs on the VCAM-1- ^EV^HB-Chip showed significant and selective enrichment of ICAM-1 (*P* < 0.001) within captured VCAM-1^+^ EC-EVs, while other endothelial cell-associated markers remained unchanged [Figure 3E].

These results collectively emphasize the versatility and specificity of the VCAM-1- ^EV^HB-Chip device in capturing VCAM-1+ cells and their associated EC-EVs. Moreover, they underscore the potential relevance of these findings in elucidating the mechanisms underlying endothelial cell activation and intercellular communication in the context of vascular inflammation and related pathologies.

## DISCUSSION

Endothelial cell activation is a pivotal event in the pathogenesis of cardiovascular and metabolic diseases, signaling the onset of inflammation and vascular dysfunction. Our study highlights an innovative use of microfluidics to capture cell-specific EC-EVs. Specifically, using anti-VCAM-1 antibodies, we demonstrate the capture of both VCAM-1^+^ endothelial cell populations, and the differential gene expression analysis of activated endothelial cells and VCAM-1^+^ EC-EVs. These findings have significant implications for our understanding of vascular inflammation and intercellular communication in the context of cardiovascular and metabolic diseases. Further, it provides initial proof of concept data to support the translation of this approach to more complex clinical samples.

The success of our study centers on the development of a sophisticated microfluidic device, bearing VCAM-1 antibodies, which enables the selective capture of VCAM-1^+^ cells and EC-EVs. This innovative approach offers several advantages over traditional techniques, particularly in its ability to target specific subpopulations of cells and EVs based on their surface markers. The use of aryl diazonium fabrication and nanoparticle-based modifications represents a notable advancement, enhancing the device's specificity and efficiency^[9,13]^. This novel technology opens up exciting possibilities for further research into the role of cell adhesion molecules like VCAM-1 in vascular pathology and metabolic disease.

Our study demonstrates the robustness of the VCAM-1- ^EV^HB-Chip in capturing VCAM-1^+^ endothelial cell populations. We found that EA.hy926 cells engineered to express TdTomato-palmitoylation-VCAM-1 exhibited significantly higher levels of soluble VCAM-1 protein in their culture supernatants compared to control cells. Furthermore, the efficient capture of these engineered cells by the VCAM-1- ^EV^HB-Chip, as opposed to IgG controls, underscores the specificity of our approach. These findings highlight the potential utility of this device in studying the dynamics of endothelial cell activation and its role in various disease states.

One of the most interesting aspects of our study is the analysis of VCAM-1^+^ EC-EVs and their mRNA cargo. We observed that HUVECs activated with pro-inflammatory TNF-α exhibited a significant increase in VCAM-1 levels in their culture supernatants. Importantly, the VCAM-1- ^EV^HB-Chip efficiently captured these TNF-α-stimulated HUVECs but not on control devices, confirming its specificity. Furthermore, the differential gene expression analysis of VCAM-1^+^ EC-EVs revealed alterations in mRNA cargo, particularly for ICAM-1, another critical endothelial cell activation marker. De Jong *et al.*^[14]^ and Seibold^[15]^ *et al.* have previously described similar enrichment of VCAM-1 and ICAM-1 transcripts inside EC-EVs, suggesting that VCAM-1^+^ EC-EVs may play a key role in intercellular signaling during vascular inflammation.

### Limitations and future directions

While our study provides valuable insights, it is not without limitations. The VCAM-1- ^EV^HB-Chip, although promising, may require further optimization to address issues related to scalability, cost-effectiveness, and across-species specificity. Additionally, the study primarily focuses on *in vitro* experiments, and further *in vivo* investigations are necessary to validate our findings in physiologically relevant settings such as plasma from patients following MI, where VCAM-1+ EVs are rapidly released into the peripheral blood.

The VCAM-1-^EV^HB -Chip is an immunoaffinity platform for the selective capture of VCAM-1+ EC-EV from heterogeneous pools; this approach, although effective, is liable to antibody concentrations and antibody efficiency in the capture of VCAM-1+ EVs. Here, we have utilized a constant antibody concentration based on our previous reports^[9,13,16]^ and a commercially available anti-VCAM-1 antibody that has been used to capture VCAM-1+ EV from plasma and to image them using transmission electron microscopy^[2]^. Different antibody concentrations and other VCAM-1 antibodies may have variable successes and would require independent validation in studies similar to those described here.

The ^EV^HB-Chip efficiently captures low abundance sub-populations from heterogeneous pools, which carry specific transmembrane proteins such as VCAM-1 for subsequent biomarker analysis using highly sensitive techniques such as digital droplet PCR. However, this limits traditional EV approaches of western blotting for characterization of captured EVs to the ^EV^HB-Chip surface, because they require very large quantities of often heterogeneous EVs for downstream analysis. How traditional EV-markers such as ALIX, HSP70, CD9, and CD63, (used here to validate the presence of EVs in the conditioned cell culture media prior to entering the VCAM-1-^EV^HB -Chip) are altered during endothelial cell activation and on VCAM-1+ EVs remain largely unknown. Future studies investigating how EV biogenesis-associated markers are altered following endothelial cell activation and on VCAM-1+ EC-EV sub-population may reveal distinct roles of VCAM-1+ EVs in vascular inflammation and in a range of pathologies with vascular dysfunction.

On-chip lysis steps and one-step PCR enable the detection of RNA cargo in low-abundance populations of EVs from heterogeneous pools^[9,13,16]^. However, this limits the use of conventional RNA quality parameters often obtained by Nanodrop and Bioanalyzer technologies. Despite the limitation, the low abundance of EV-transcripts here shows the presence of thousands of positive droplets in digital PCR for ICAM-1 versus transcripts which are ordinarily very high inside the cell such as Gapdh and Actb. We have previously shown the enrichment of miRNA-126-3p and miRNA-126-5p inside endothelial cell-derived EVs following inflammatory activation^[2]^. Whether VCAM-1+ EVs carry a specific miRNA-cargo or differential levels of miRNA-126-3p and miRNA-12-5p is yet to be determined, but this highlights another functional advantage of the VCAM-1- ^EV^HB-Chip to detect very low abundant EV subpopulations and their RNA cargo which may extend to other non-coding RNAs.

The VCAM-1- ^EV^HB-Chip efficiently captures VCAM-1+ EC-EVs, but subsequent release of the captured VCAM-1+ EVs remains unexplored. Our recent aryl-diazonium salts coating modifications to the ^EV^HB-Chip^[9]^ may enable the elution of captured VCAM-1+ EC-EVs for subsequent investigation and analysis. We hypothesize that the use of NHS-biotin linkers with disulfide bonds may enable the subsequent release of captured EVs from the device with a reducing agent similar to dithiothreitol (DTT), beta-mercaptoethanol (BME), or N-Acetyl cysteine.

Besides the ongoing VCAM-1- ^EV^HB-Chip developments and potential applications in clinical studies, the specific mechanisms by which VCAM-1^+^ EC-EVs mediate intercellular communication and enriched ICAM-1 mRNA transcripts warrant deeper exploration.

## CONCLUSION

In conclusion, our study leverages an innovative microfluidic technology to investigate endothelial cell activation and the role of VCAM-1 in vascular inflammation. We demonstrate that the efficient capture of VCAM-1^+^ endothelial cell populations and their associated extracellular vesicles has the potential to shed light on the early molecular events that precede cardiovascular and metabolic diseases. While this research represents a significant step forward, ongoing efforts are needed to fully elucidate the intricacies of endothelial cell activation and its impact on disease progression. Our findings provide a foundation for future studies that may uncover novel therapeutic targets and interventions for these prevalent and debilitating conditions, ultimately advancing our ability to combat cardiovascular and metabolic diseases.

## DECLARATIONS

### Availability of data and materials

The data and materials supporting the findings of this study are available upon reasonable request from the corresponding authors.

### Copyright

© The Author(s) 2024.
