# Supplementary material for: The isolation of VCAM-1+ endothelial cell-derived extracellular vesicles using microfluidics
Source: Extracell Vesicles Circ Nucl Acids. 2024 Feb 8;5(1):83–94. doi: 10.20517/evcna.2023.51 (PMC11648473; doi:10.20517/evcna.2023.51)
Supplement: Supplementary file 1 [file evcna-5-1-83-SupplementaryMaterials.pdf]

**Naveed Akbar[1,2], Evelyn Grace Luciani[2], Raheel Ahmad[2], Dasol Lee[2], Sara Veiga[2], Daniel Christopher Rabe[2], Shannon Leigh Stott[2]**

[1]Division of Cardiovascular Medicine, Radcliffe Department of Medicine, University of Oxford, Oxford OX3 9DU, UK.

[2]Center for Engineering in Medicine & Surgery, Massachusetts General Hospital Cancer Center, Harvard Medical School, Charlestown, MA 02129, USA.

**Correspondence to**: Dr. Naveed Akbar, Division of Cardiovascular Medicine, Radcliffe Department of Medicine, University of Oxford, John Radcliffe Hospital, West Wing, Headley Way, Oxford OX3 9DU, UK. E-mail: Naveed.Akbar@cardiov.ox.ac.uk;Dr. Shannon Leigh Stott, Center for Engineering in Medicine & Surgery, Massachusetts General Hospital Cancer Center, Harvard Medical School, 149 13th Street, Charlestown, MA 02129, USA. E-mail: sstott@mgh.harvard.edu

**Supplementary Figure 1.** Uncropped Western Blot Images

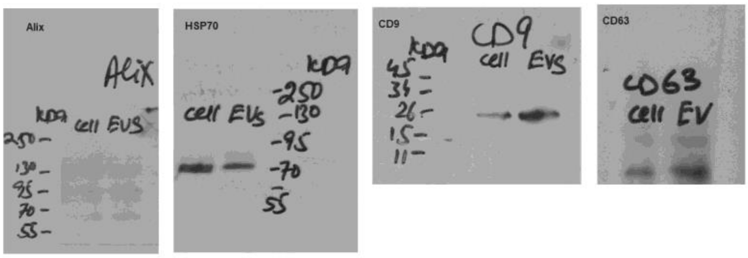


**Supplementary Table 1. RT-qPCR Primers**

| Probe Name | Label/Tag | Sequence/Product Code | Concentration |
|---|---|---|---|
| GAPDH | HEX | Hs.PT39a22214836 | 40x |
| ACTB | HEX | HS.PT.39a22214847 | 40x |
| KDR | FAM | Hs.PT.58.40453650 | 20x |
| VCAM-1 | FAM | Hs.PT.58.20405152 | 20x |
| CAV.1 | HEX | Hs.PT.58.470742 | 20x |
| ICAM-1 | HEX | Hs.PT.58.4962347 | 20x |
| MEOX2 | HEX | Hs.PT.58.20106818 | 20x |

| | | | |
|---|---|---|---|
| EPCAM | FAM | Hs.PT.58.4797664 | 20x |
| FABP4 | FAM | Hs.PT.56a.3615957 | 20x |
| ENG | FAM | Hs.PT.58.19487865 | 20x |
| THBD | HEX | Hs.PT.58.3285240 | 20x |
| EGLF7 | FAM | Hs.PT.58.4610082 | 20x |
| CDH5 | FAM | Hs.PT.56a.27589054 | 20x |
| PECAM-1 | HEX | Hs.PT.58.4732035 | 20x |
| CD36 | HEX | Hs.PT.58.4610082 | 20x |
| MCAM | HEX | Hs.PT.58.39570895 | 20x |
| TIE1 | FAM | Hs.PT.58.40334940.g | 20x |
